# Supplementary material for: Effect of Feed Supplementation with Tripotassium Citrate or Sodium Chloride on the Development of Urinary Calcium Oxalate Crystals in Fattening Pigs
Source: Vet Sci. 2022 Nov 6;9(11):614. doi: 10.3390/vetsci9110614 (PMC9699466; doi:10.3390/vetsci9110614)
Supplement: Supplementary file 1 [file vetsci-09-00614-s001.zip › vetsci-1964997-supplementary.pdf]

Table S1. General composition (minimum levels as mentioned on the leaflet) of the five different mash feeds that were used on the farm and provided to the pigs from the start until the end of the fattening period

	Feed				
	1	2	3	4	5
Provided to pigs of … kg	20-40	40-55	55-70	70-95	95-slaughter
Crude protein (%)	16.3	15.5	15	14.2	14.0
Crude fat (%)	4.7	4.2	4.5	4.4	4.2
Crude fiber (%)	4.7	4.5	5.0	4.8	4.8
Crude ash (%)	4.8	4.5	4.4	4.5	4.4
Total P (%)	0.40	0.40	0.37	0.37	0.37
Na (%)	0.18	0.16	0.20	0.18	0.18
Ca (%)	0.65	0.65	0.60	0.61	0.62
Vit A (IU/kg)	15000	6500	6500	6500	6500
Vit D3 (IU/kg)	2000	2000	2000	2000	2000
Vit E (ppm)	80	100	100	100	100
Lysine (%)	1.07	1.00	0.95	0.89	0.86
Methionine (%)	0.38	0.33	0.31	0.30	0.29

Table S2. Biochemical and microbiological analysis of the drinking water used for the fattening pigs on the farm. The drinking water originated from the public supply, and a sample was taken from a drinking nipple in the pen.

Parameter	Value
Biochemical analysis	
pH	7.85
Nitrate (mg/L)	17.86
Nitrite (mg/L)	0.01
Ammonium (mg/L)	0.46
Bicarbonate (mg/L)	330
Calcium (mg/L)	133
Magnesium (mg/L)	9.13
Sodium (mg/L)	44.02
Potassium (mg/L)	6.20
Phosphorus (mg/L)	0.07
Boron (mg/L)	0.10
Iron (mg/L)	1.02
Manganese (mg/L)	0.05

Copper (mg/L)	0.04
Zinc (mg/L)	0.31
Microbiological analysis	
Bacterial count at 22°C (CFU/mL)	234
Bacterial count at 37°C (CFU/mL)	28
Coliform bacteria (CFU/100mL)	0
Escherichia coli (CFU/100mL)	0
Enterococci (CFU/100mL)	0
